# Population-Based Study of Rare Coding Variants in *NR5A1*/SF-1

**DOI:** 10.1210/jendso/bvae178

**Published:** 2024-10-23

**Authors:** Chrysanthi Kouri, Raina Y Jia, Katherine A Kentistou, Eugene J Gardner, John R B Perry, Christa E Flück, Ken K Ong

**Affiliations:** Department of Pediatrics, Pediatric Endocrinology, Diabetology and Metabolism, Inselspital, Bern University Hospital, University of Bern, 3010 Bern, Switzerland; Department for BioMedical Research, University of Bern, 3008 Bern, Switzerland; Graduate School for Cellular and Biomedical Sciences, University of Bern, 3012 Bern, Switzerland; MRC Epidemiology Unit, Institute of Metabolic Science, University of Cambridge, Cambridge CB2 0QQ, UK; MRC Epidemiology Unit, Institute of Metabolic Science, University of Cambridge, Cambridge CB2 0QQ, UK; MRC Epidemiology Unit, Institute of Metabolic Science, University of Cambridge, Cambridge CB2 0QQ, UK; MRC Epidemiology Unit, Institute of Metabolic Science, University of Cambridge, Cambridge CB2 0QQ, UK; Department of Pediatrics, Pediatric Endocrinology, Diabetology and Metabolism, Inselspital, Bern University Hospital, University of Bern, 3010 Bern, Switzerland; Department for BioMedical Research, University of Bern, 3008 Bern, Switzerland; MRC Epidemiology Unit, Institute of Metabolic Science, University of Cambridge, Cambridge CB2 0QQ, UK; Department of Paediatrics, University of Cambridge, Cambridge CB2 0QQ, UK

**Keywords:** steroidogenic factor 1 (SF-1/NR5A1), differences of sex development (DSD), obesity, whole exome sequencing (WES), UK Biobank (UKBB)

## Abstract

**Background:**

Steroidogenic Factor 1/Nuclear Receptor Subfamily 5 Group A Member 1 (SF-1/*NR5A1*) is critical for the development and function of sex organs, influencing steroidogenesis and reproduction. While rare deleterious *NR5A1*/SF-1 variants have been identified in individuals with various differences of sex development (DSD), primary ovarian insufficiency, and infertility, their impact on the general population remains unclear.

**Methods:**

We analyzed health records and exome sequencing data from up to 420 162 individuals (227 858 women) from the UK Biobank study to assess the impact of rare (frequency < 0.1%) predicted deleterious *NR5A1*/SF-1 variants on age at menopause and 26 other traits.

**Results:**

No carriers of rare protein truncating variants in *NR5A1*/SF-1 were identified. We found that the previously reported association of rare deleterious missense *NR5A1*/SF-1 variants with earlier age at menopause is driven by variants in the DNA binding domain (DBD) and ligand binding domain (LBD) (combined test: beta = −2.36 years/allele, [95% CI: 3.21, −1.51], N = 107 carriers, *P* = 4.6 × 10^−8^). Carriers also had a higher risk of adult obesity (OR = 1.061, [95% CI: 1.003, 1.104], N = 344, *P* = .015), particularly among women (OR = 1.095 [95% CI: 1.034, 1.163, *P* = 3.87 × 10^−3^], N = 176), but not men (OR = 1.019, [95% CI: 0.955, 1.088], *P* = .57, N = 168).

**Conclusion:**

Deleterious missense variants in the DBD and LBD likely disrupt *NR5A1*/SF-1 function. This study broadens the relevance of deleterious *NR5A1*/SF-1 variants beyond rare DSDs, suggesting the need for extended phenotyping and monitoring of affected individuals.

Rare deleterious mutations in Steroidogenic Factor 1/Nuclear Receptor Subfamily 5 Group A Member 1 (SF-1/*NR5A1*) are prevalent among individuals with differences of sex development (DSD) presenting with a wide spectrum of phenotype ranging from mild to severe. They occur mostly in heterozygous but also in homozygous state [1-4]. But, *NR5A1*/SF-1 variants can also be identified in individuals without DSD, including healthy carriers, infertile men, or women with primary ovarian insufficiency (POI) [4-8]. Broader health impacts of *NR5A1*/SF-1 variations are possible, given the key role of this gene in adrenal and reproductive development and function [9]. So far, our knowledge regarding the effects of *NR5A1*/SF-1 variants beyond DSD is limited.

Early studies on SF-1 knockout (KO) mice hinted at the involvement of SF-1 in energy balance, as evidenced by the compromised structure of the ventromedial hypothalamic nucleus (VMH) [10, 11], a recognized regulator of energy homeostasis and body weight [12-15]. SF-1 neurons, which are a subset of neurons primarily located in the VMH, are regulated by various factors such as nutrients (eg, glucose) and hormones (eg, leptin and insulin) [16]. SF-1 KO mice exhibited late-onset obesity due to decreased energy expenditure rather than hyperphagia, establishing them as potential model for hypothalamic late-onset obesity [17].

Subsequent investigations [18, 19] focused on the role of leptin in SF-1 neurons and obesity. Conditional KO mice lacking leptin receptors (LepRs) in SF-1 neurons, displayed obesity, particularly under a high-fat diet condition. The idea of a distinct functional subsets of leptin-activated and leptin-inhibited SF-1 neurons has been proposed [20, 21]. Moreover, mice with genetic modifications affecting various elements in SF-1 neurons displayed a modified metabolic phenotype, primarily evident under high-fat diet conditions, rather than a standardized nutritional balanced diet [16]. These observations suggested that SF-1 neurons may play a role in metabolic adaptation, particularly during the initial phases of obesity.

Optogenetic and chemogenetic studies have further elucidated the functions of SF-1 neurons in energy balance and glucose regulation. Optogenetic stimulation showed that SF-1 neurons exert differential effects depending on the frequency of activation [22]. Low-frequency activation of SF-1 neurons in mice resulted in lower food intake and less interest in food, shown by a decrease in time the mice spent near their food. Similarly, chemogenetic activation of SF-1 neurons led to a reduction in food intake and an increase in energy expenditure and fat oxidation in fasted mice [23], while inhibition of SF-1 neurons led to increase in cumulative food intake in mice with ad libitum feeding [24]. Conversely, in another study, optogenetic activation of SF-1 neurons resulted in diabetes-range hyperglycemia [25]. Conflicting results across these studies support the idea that glucose regulation by SF-1 neurons may be mediated by distinct subsets of neurons, leading to either an increase in insulin sensitivity or hyperglycemia.

While numerous studies in mouse models establish the role of SF-1 in metabolic regulation, limited data are available for DSD individuals with rare deleterious *NR5A1*/SF-1 variants and their association to obesity or overweight [26-30]. In one study in the Han Chinese population, the specific *NR5A1*/SF-1 variant p.Gly146Ala was associated with type 2 diabetes [31]. Data on metabolic health outcomes in individuals with rare *NR5A1*/SF-1 variants are limited due to the rarity of the disorder and the fact that, so far, the clinical focus was primarily on DSD (eg, pubertal and fertility issues) [4]. Thus, the younger age of studied individuals may lead to the oversight and under-reporting of late-onset obesity or other metabolic health issues in these individuals.

Apart from SF-1 involvement in metabolism, spleen anomalies (eg, spleen hypoplasia) are reported in a significant number of individuals with rare *NR5A1*/SF-1 variants [4, 30, 32, 33], supported also by mouse model data [34].

These collective findings suggest a potential role of SF-1 in broader aspects of human health beyond reproduction. To address this question, we analyzed multiple sources of health record data and exome sequencing data of up to 420 162 individuals from the population-based UK Biobank study. We investigated the impact of rare (frequency < 0.1%) predicted deleterious *NR5A1*/SF-1 variants on potentially relevant traits with reproduction, puberty timing, metabolism, and spleen function.

## Methods

### UK Biobank Data Processing and Quality Control

To conduct rare variant burden analyses, we obtained whole exome sequencing (WES) data for 454 787 individuals provided by the UK Biobank (UKBB) study [35]. Individuals were excluded from subsequent analyses if they exhibited excessive heterozygosity, had autosomal variant missingness on genotyping arrays of ≥5%, or were not encompassed within the subset of phased samples as defined by Bycroft et al [36]. Additionally, we excluded individuals who did not have a broadly European genetic ancestry, resulting in a final WES dataset of 420 162 individuals. Additional exclusions occurred during the analysis of specific phenotypes due to incomplete data.

We used the UKBB Research Analysis Platform (RAP; https://ukbiobank.dnanexus.com/) for variant quality control and annotation. The RAP is a cloud-based computing environment and central data repository for UKBB WES and phenotypic data. Customized applets designed for the RAP facilitated additional quality control on exome sequencing data [37], expanding on the procedures outlined in Backman et al [35]. Using provided population-level Variant Call Format (VCF) files, we first split and left-corrected multi-allelic variants into separate alleles using “bcftools norm.” Next, we performed genotype-level filtering using “bcftools filter” separately for single nucleotide variants (SNVs) and insertions/deletions (InDels) using a missingness-based approach. With this approach, SNV genotypes with depth < 7 and genotype quality < 20 or InDel genotypes with a depth < 10 and genotype quality < 20 were set to missing (ie, ./.). We further tested for an expected alternate allele contribution of 50% for heterozygous SNVs using a binomial test; SNV genotypes with a binomial test *P* value ≤ 1 × 10^−3^ (indicative of a genotype calling error or somatic mutation) were set to missing. Following genotype-level filtering we recalculated the proportion of individuals with a missing genotype for each variant and filtered out all variants with a missingness value > 50%.

Following that, we annotated variants by predicting their functional consequences using ENSEMBL Variant Effect Predictor (VEP) v104 [38]. We applied the “–everything” flag and activating plugins for REVEL [39], CADD [40], and LOFTEE [41]. For each variant, we assigned priority to a specific ENSEMBL transcript based on whether the annotated transcript was protein-coding, MANE select v1.0 [42], or the VEP Canonical transcript, respectively. The consequence for each individual variant was determined by prioritizing severity according to VEP's definitions. After the annotation process, we consolidated stop gained, frameshift, splice acceptor, and splice donor variants into a unified category known as *protein truncating variants* (PTVs). The consequences for missense and synonymous variants were determined following the definitions provided by VEP. Subsequently, only autosomal or chrX variants within ENSEMBL protein-coding transcripts and within transcripts covered by the UKBB WES assay were retained for subsequent burden testing.

### Exome-Wide Association Analyses in the UK Biobank

Here, we used the previously reported impact of rare predicted deleterious variants in the *NR5A1/*SF-1 gene on age at natural menopause (ANM) in this cohort [43], to optimize our definition of deleterious variant class for association testing. We then explored the effect of high-confidence PTVs and missense variants with CADD score ≥25 on other potentially relevant traits in UKBB: age at menarche in women (AAM), age at voice breaking in men (AVB), birth weight, blood pressure (diastolic and systolic), body fat percentage, body mass index (BMI), childlessness, circulating IGF-1 (insulin-like growth factor 1) levels, glucose levels, glycated hemoglobin (HbA1c), high-density lipoprotein (HDL) cholesterol, height, low-density lipoprotein (LDL) cholesterol, number of live births, obesity (BMI >30 kg/m^2^), red blood cell count, relative adiposity in childhood, SHBG (sex hormone binding globulin) levels, testosterone levels, triglycerides, type 2 diabetes, and waist hip ratio (Supplementary Table S1) [44].

To conduct rare variant burden tests, we used a custom implementation of BOLT-LMM v2.3.6 [45] for the RAP. BOLT-LMM requires 2 main inputs: (i) genotypes at variants with a minor allele count > 100 obtained from genotyping arrays to construct a null linear mixed effects model; and (ii) a larger set of variants collapsed on ENSEMBL transcript to perform association tests. For the former, we extracted genotyping data available on the RAP, limiting it to the same set of individuals used for rare variant association tests. For the latter, since BOLT-LMM expects imputed genotyping data rather than per-gene carrier status, we created dummy genotype files where each variant represented one gene, and individuals with a qualifying variant within that gene were coded as heterozygous, irrespective of the number of variants they carried in that gene.

We generated dummy genotype files for 3 overlapping variant classes: (i) high-confidence PTVs defined by LOFTEE; (ii) missense variants with CADD ≥ 25; and (iii) damaging variants, which included both high-confidence PTVs and missense variants with CADD ≥ 25. For each phenotype tested, BOLT-LMM was executed with default parameters, with the inclusion of the “lmmInfOnly” flag. To obtain association statistics for individual markers, we provided all 26 657 229 markers, regardless of their filtering status, as input to BOLT-LMM. All tested phenotypes were treated as continuous traits or binary traits, adjusted for age, age squared (age^2^), sex, the first 10 genetic principal components as calculated in Bycroft et al [36], and WES batch as a categorical covariate (50k, 200k, or 450k).

### Phenotype Derivation

For the phenotype derivation and processing refer to Supplementary Table S1 [44].

### SF-1 Protein Domain-Specific Analyses

To explore how rare deleterious missense variants disrupt SF-1 function, we next categorized variants by protein domain. Briefly, we included predicted deleterious missense variants with CADD score ≥ 25 with MAF < 0.1% which map to either of the 2 main functional domains of SF-1 protein (DNA binding domain [DBD] and ligand binding domain [LBD]), as defined by UniProt. We implemented a generalized linear model (GLM) using the Python package “statsmodels” [46]. For the domain-level burden test, the number of variant alleles across the 2 domains was summed up into a single score under a simple additive model. This score was used as a predictor of the traits in a three-step regression. All traits assessed with BOLT-LMM were included in the GLM burden test, and in addition, we expanded the analysis to encompass additional traits, including spleen volume, platelet count, and reticulocyte count (Supplementary Table S1) [44]. For all association tests we corrected for age, age^2^, the first 10 genetic principal components provided by Bycroft et al [36], and study participant WES batch as a categorical covariate.

All analyses and data manipulations were conducted in R (v4.1.2) and in the UKB Research Analysis Platform (RAP; https://ukbiobank.dnanexus.com/).

## Results

### Rare Deleterious *NR5A1*/SF-1 Variants and Earlier Age at Natural Menopause

**Figure 1.**
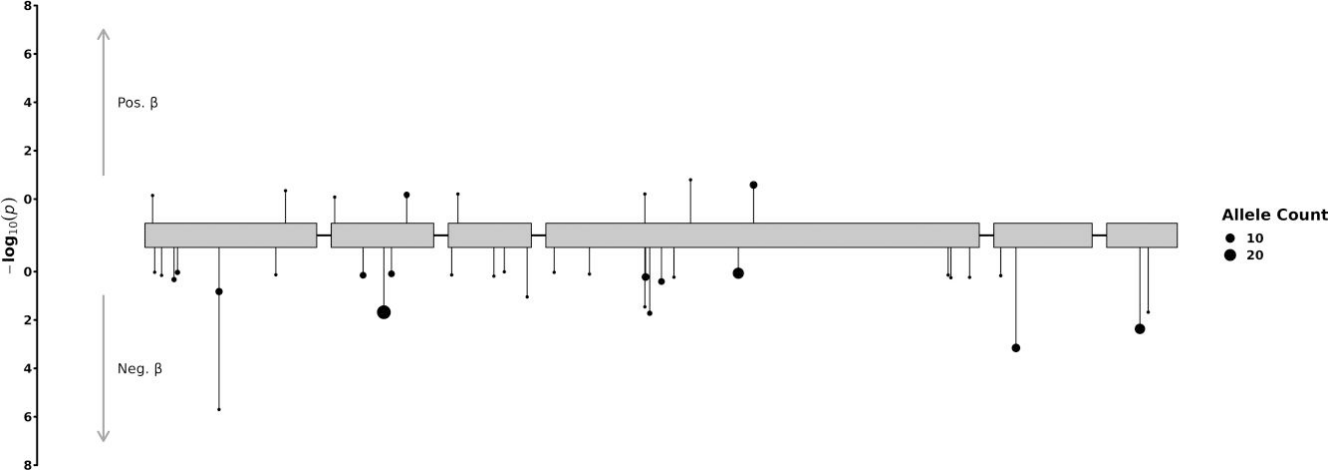
Exome associations between *NR5A1*/SF-1 gene with age at natural menopause in the UKBB. Included were *NR5A1*/SF-1 variants with MAF smaller than 0.1% and annotated as missense variants with a high CADD score ≥ 25, distributed across exons of *NR5A1* gene. Each variant is depicted as a separate line, extending to its association *P* value (−log10), with the direction indicating the effect on age at natural menopause in carriers of the alternate allele. The point size corresponds to the respective variant's allele count, as indicated in the figure legend.

Using WES data from 227 858 women of European genetic ancestry, we confirmed that individuals carrying rare deleterious missense variants (with CADD score ≥ 25) in the *NR5A1/SF-1* gene had earlier ANM (beta = −1.76 years, [−2.50, −1.01], *P* = 3.80 × 10^−6^, N = 131) (Fig. 1, Supplementary Table S2) [44]. Notably, we did not identify any carrier of rare PTVs alleles in *NR5A1/*SF-1.

### Trait Associations Beyond Reproduction

**Figure 2.**
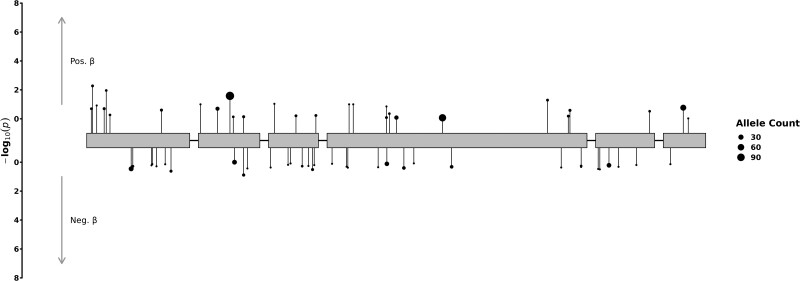
Exome associations between *NR5A1*/SF-1 gene with obesity in the UKBB. Included were *NR5A1*/SF-1 variants with MAF smaller than 0.1% and annotated as missense variants with a high CADD score ≥ 25, distributed across exons of NR5A1 gene. Each variant is depicted as a separate line, extending to its association *P* value (−log10), with the direction indicating the effect on obesity in carriers of the alternate allele. The point size corresponds to the respective variant's allele count, as indicated in the figure legend.

Using the same variant mask of rare deleterious missense variants with CADD score ≥ 25 (Supplementary Table S3) [44] to explore associations with additional traits (Supplementary Table S1) [44], we observed nominal associations of the *NR5A1/SF-1* gene with obesity (odds ratio [OR] = 1.041, [1, 1.078], *P* = .046, N = 472) (Fig. 2), HbA1c levels (beta = .54, [0.11, 0.97], *P* = .013, N = 451), HDL (beta = .04, [0.012, 0.073], *P* = 6.5 × 10^−3^, N = 395), circulating IGF-1 levels (beta = −.48, [−0.95, 0], *P* = .05, N = 415) and systolic blood pressure (beta = .46, [−1.41, 2.32], *P* = .03, N = 442) (Supplementary Table S2) [44]. Surprisingly, we found no association between this variant mask with other expected reproductive traits, eg, puberty timing in either sex (Supplementary Table S2) [44].

### Individual Variant-Level Analyses

Next, we conducted individual rare deleterious *NR5A1/SF-1* variant-level association tests with ANM. We found 7 variants that showed nominally significant individual associations (*P* ≤ .05) with earlier ANM. Notably, one of these variants (c.43G>A; p.Val15Met, Allele count (AC) = 1) has previously been reported in rare cases of 46,XY sex reversal [47], and premature ovarian insufficiency [48] (Supplementary Table S4) [44]. A second variant (c.721C>T; Arg241Trp, AC = 2) has been also reported in a rare case of 46,XY sex reversal [49], (Supplementary Table S4) [44]. Lastly, a third variant (c.1063G>A; p.Val355Met, AC = 135) has been reported in a boy with bilateral anorchia, micropenis, and progressive testicular degeneration. His mother, who also carried this variant, had 2 spontaneous miscarriages, and had left ovariectomy and removal of the corresponding fallopian tube due to ovarian cysts at the age of 22 (Supplementary Table S4) [44, 50].

### Rare Deleterious Missense Variants Within SF-1 Protein Domains

To explore how rare deleterious missense *NR5A1*/SF-1 variants associated with ANM disrupt its protein function, we categorized them by the SF-1 protein domains. Only 3 variants (n = 24 carriers) were found in the hinge region of the SF-1 protein, and none showed an association with ANM (all *P* > .05). Thus, we focused only on variants in the DBD and LBD.

Using GLM domain-level burden testing, we found that carriers of rare deleterious missense *NR5A1/*SF-1 variants in each domain had earlier ANM (DBD: beta = −4.03 years, [−5.71, −2.34], *P* = 2.80 × 10^−6^, N = 27; LBD: beta = −1.80, [−2.78, −0.82], *P* = 3.18 × 10^−4^, N = 80). We also tested if considering both DBD and LBD simultaneously increased our strength of association with ANM and found that the association between *NR5A1/*SF-1 and ANM was strengthened (beta = −2.36, [−3.20, −1.51], *P* = 4.6 × 10^−8^, N = 107). This combined analysis demonstrates a more robust and significant relationship between *NR5A1*/SF-1 variants and ANM compared to the domain-agnostic test, underscoring the importance of domain-specific effects in influencing NR5A1 function and menopausal timing.

We used this “domains” mask of grouped variants in DBD and LBD to search for *NR5A1/*SF-1 gene-level associations with other traits beyond ANM, using GLM burden testing. Associations with domain-unrestricted variants are shown for comparison in Supplementary Table S5 [44]. Our analysis revealed that rare deleterious variants in the DBD and LBD of the *NR5A1*/SF-1 gene were associated with a higher risk of obesity (sex combined: OR = 1.061, [1.01, 1.106], *P* = .015, N = 344), particularly in women (OR = 1.057, [1.034, 1.163], *P* = 3.87 × 10^−3^, N = 176), but not in men (OR = 1.019, [0.955, 1.088], *P* = .57, N = 168) (Supplementary Table S5) [44]. Notably, 32.3% of women who carried rare deleterious missense NR5A1/SF-1 variants located in the DBD and LBD had obesity, compared to 23% of noncarriers. Additionally, we found a positive association of these variants with higher platelet count (beta = 8.55 × 10^9^ cells/L, [2.36 × 10^9^, 14.73 × 10^9^], *P* = 6.8 × 10^−3^, N = 334) in both sexes (Supplementary Table S5) [44].

## Discussion

Here we present the results of an exome-wide association study assessing the collective impact of rare (frequency < 0.1%), predicted deleterious *NR5A1*/SF-1 variants in individuals from the population-based UKBB study. We aimed to understand the potential associations of these variants with traits putatively linked to the function of *NR5A1;* a gene known for its pivotal role in reproductive development and function, yet not thoroughly characterized in humans for effects on other health outcomes such as metabolism. A similar analysis reported an association of rare *NR5A1*/SF-1 variants with earlier age at menopause in women in the same UKBB cohort [43]. In the current study, we found that this association is driven by deleterious variants that specifically code the DBD and LBD of the protein. Through further protein domain association testing, we identified that women who carry rare deleterious missense *NR5A1*/SF-1 variants located in the DBD and LBD also have an elevated risk of obesity.

These consistent findings on earlier ANM align with the known role of rare deleterious *NR5A1*/SF-1 variants in POI in women [2, 5, 7, 51]. SF-1 regulates crucial genes of ovarian development, steroidogenesis, growth and maturation of follicles, the disruption of which can lead to ovarian dysfunction [5]. However, incomplete penetrance, together with the suggested role of oligogenic contribution [4, 52, 53] to the phenotype of individuals with *NR5A1*/SF-1 variants, underscores the complexity of genotype-phenotype correlations. Further investigations are needed to identify potential modifying factors that contribute to the variable clinical outcomes associated with *NR5A1*/SF-1 variants in the context of ovarian function. This understanding will be crucial for refining risk assessment and facilitating more accurate genetic counseling for affected individuals and their families.

The observed association of deleterious *NR5A1*/SF-1 variants with obesity supports the proposed involvement of SF-1 in regulating energy metabolism, beyond its role in reproductive function. Previous research suggested a role for SF-1 in energy balance, as evidenced by studies on SF-1 KO mice and the regulatory role of SF-1 neurons in the VMH [10, 11]. Subsequent investigations into the impact of SF-1 on leptin signaling and the development of obesity, especially under high-fat diet conditions, further informed on the multifaceted role of SF-1 in metabolic adaptation [16-24]. Optogenetic and chemogenetic studies provided additional insights, revealing the diverse effects of SF-1 neurons on feeding behavior, energy expenditure, and glucose regulation [22-25].

There is limited previous evidence on the possible link between rare *NR5A1*/SF-1 variants and obesity in humans. Previous reports are limited to individuals with DSD [26-30], revealing a critical gap in translating mouse model findings to human populations. This scarcity is exacerbated by challenges in studying metabolic health outcomes in individuals with rare *NR5A1*/SF-1 variants due to the few numbers [26-30]. In individuals with rare *NR5A1*/SF-1 variants and DSD, the clinical focus is primarily on reproductive and urogenital issues during younger ages, and the majority of affected individuals have a 46,XY karyotype [1, 4]. Women with rare *NR5A1*/SF-1 variants and DSD or POI are less frequently reported [3, 5, 7], which limits the understanding of the sex-specific role of SF-1. In our study the association of rare *NR5A1*/SF-1 variants with obesity particularly in women, could be linked to sex-specific hormonal dysregulation, potentially rendering women with these variants more susceptible to obesity compared to men. Our study not only emphasizes the genetic association between rare predicted deleterious *NR5A1*/SF-1 variants and obesity but also underscores the need for more comprehensive research to bridge the translational gap between mouse models and humans, younger and older age, as well as sex differences.

In this study, we also observed modestly higher platelet counts in carriers of deleterious missense *NR5A1*/SF-1 variants. Spleen anomalies including hypoplasia are reported in some individuals with rare *NR5A1*/SF-1 variants. While *NR5A1* carriers had higher platelet counts, spleen volume was measured only in a subset of UKBB and only 29 carriers had this data. Thrombocytosis (high platelet count > 450 000/μL) [54] has previously been reported in individuals with DSD due to *NR5A1*/SF-1 variants, possibly indicating spleen dysfunction [4, 33, 55]. In combination with our findings, we suggest that these variants affect spleen function, due to the impaired activity of SF-1 during spleen development [30, 32, 33].

It is noteworthy that we did not find carriers of rare protein truncating variants in *NR5A1*/SF-1 in the UKBB population. This likely indicates the gene's critical importance and its conservation within human populations. While there are no known mutation hotspots in the SF-1 protein, our study revealed a concentration of rare deleterious missense *NR5A1*/SF-1 variants in the DBD and LBD of the SF-1 protein. This observation is consistent with findings from other studies, where the majority of reported rare deleterious variants are commonly located in these 2 domains, which constitute a significant portion of the protein [1, 4].

We acknowledge limitations of our study. Firstly, consistent with other large-scale exome sequencing studies, we are unable to validate and replicate our findings in an independent cohort. While the association with ANM was our primary hypothesis, associations with other potentially relevant traits should be considered as exploratory and should be confirmed in other large cohorts or patient series. Additionally, our reliance on in silico predictions to assess the functional implications of rare *NR5A1*/SF-1 variants underscores the need for future extensive experimental characterization to accurately assess their impact on protein function. Furthermore, we acknowledge the influence of “healthy volunteer bias” in the UK Biobank [56], which could attenuate disease associations.

Despite these limitations, our study proposes links between rare predicted deleterious missense *NR5A1*/SF-1 variants with obesity and adverse metabolic outcomes. It is one of the first studies investigating the impact of rare *NR5A1*/SF-1 variants in the general population. Further research is needed to identify the possible sex hormone related or independent pathways linking *NR5A1*/SF-1 to obesity risk. Meanwhile, our study suggests the need for clinical awareness and characterization to detect and mitigate possible adverse health metabolic outcomes in individuals with rare *NR5A1*/SF-1 variants.

## Data Availability

Original data generated and analyzed during this study are included in this published article or in the data repositories listed in References. UK Biobank data are available on application (https://www.ukbiobank.ac.uk/enable-your-research/register).

## Abbreviations

ANM: age at natural menopause
DBD: DNA binding domain
DSD: differences of sex development
GLM: generalized linear model
HbA1c: glycated hemoglobin
HDL: high-density lipoprotein
IGF-1: insulin-like growth factor 1
KO: knockout
LBD: ligand binding domain
NR5A1: Nuclear Receptor Subfamily 5 Group A Member 1
OR: odds ratio
PTV: protein truncating variant
RAP: UKBB Research Analysis Platform
SF-1: steroidogenic factor 1
SNV: single nucleotide variation
UKBB: UK Biobank
VEP: Variant Effect Predictor
VMH: ventromedial hypothalamic nucleus
WES: whole exome sequencing
